# Dietary Stevioside Supplementation Alleviates Lipopolysaccharide-Induced Intestinal Mucosal Damage through Anti-Inflammatory and Antioxidant Effects in Broiler Chickens

**DOI:** 10.3390/antiox8120575

**Published:** 2019-11-21

**Authors:** Jingle Jiang, Lina Qi, Zengpeng Lv, Song Jin, Xihui Wei, Fangxiong Shi

**Affiliations:** 1 National Experimental Teaching Demonstration Center of Animal Science, College of Animal Science and Technology, Nanjing Agricultural University, Nanjing 210095, China; 2 Changzhou Animal Disease Control Center, Bureau of Agriculture and Rural Affairs of Changzhou, Changzhou 213003, China

**Keywords:** stevioside, anti-inflammatory, antioxidant, intestinal mucosae, broiler, LPS

## Abstract

The study was conducted to investigate the effects of dietary stevioside (STE) supplementation on the lipopolysaccharide (LPS)-induced intestinal mucosal damage of broiler chickens. A total of 192 one-day-old male Ross 308 broiler chicks were randomly divided into four treatments: (1) basal diet (CON); (2) basal diet supplemented with 250 mg/kg stevioside (STE); (3) basal diet + LPS-challenge (LPS); (4) basal diet supplemented with 250 mg/kg stevioside + LPS-challenge (LPS + STE). LPS-challenged groups received an intraperitoneal injection of LPS at 17, 19 and 21 d, whereas the CON and STE groups received a saline injection. The results showed that dietary STE supplementation normalized LPS-induced changes in protein expression of p-NF-κB and p-IκBα, mRNA expression of inflammatory genes (TLR4, NF-κB, and IFN-γ), tight junction-related genes (CLDN2, OCLN, and ZO-1), and antioxidant genes (Nrf2 and HO-1). LPS-induced decreases in serum diamine oxidase (DAO) level, villus height-to-crypt depth ratio, apoptotic index, and protein expression of proliferating cell nuclear antigen (PCNA) were reversed with dietary STE supplementation. Additionally, STE supplementation ameliorated the redox damage by reducing malondialdehyde (MDA) content and increasing total antioxidant capacity (T-AOC) and antioxidant enzyme activity. In conclusion, dietary stevioside supplementation could alleviate LPS-induced intestinal mucosal damage through anti-inflammatory and antioxidant effects in broiler chickens.

## 1. Introduction

The main function of intestinal mucosae is to maintain intestinal homeostasis and to prevent pathogenic microorganism invasion. In poultry production, intestinal mucosal damage is a common disease in broiler chickens. Intestinal mucosal damage is attributable to various pathogenic factors, including environmental stress, bacterial infection, immunological stress and oxidative stress [1,2]. Avian pathogenic E. coli (APEC) is a major pathogen in the poultry industry. Lipopolysaccharide (LPS), an endotoxin secreted by APEC, is able to activate toll-like receptor 4 (TLR4). The activation of TLR4 triggers the phosphorylation of nuclear factor kappaB (NF-κB), and eventually promotes the synthesis of pro-inflammatory cytokines and induces oxidative stress [3,4,5]. The immune response of LPS administration is proven to be similar to avian pathogenic E. coli infection on the epithelial cell of chickens [6]. Previous studies have shown that LPS challenge impairs the integrity, permeability, and oxidative status of the intestine in broiler chickens [7,8,9]. Thus, LPS has been widely applied to establish a model of intestinal mucosal damage in broiler chickens [2,7,8,9,10]. It is necessary to explore feed additives with therapeutic potential for disrupted intestinal homeostasis of LPS-challenged in broilers.

Stevioside (STE) is a natural diterpenoid glycoside extracted from the *Stevia rebaudiana* (Bertoni), which has been proven to be safe in the food industry [11]. A previous study has suggested that STE exerts no harmful effects in the chicken diet with a dose of 667 mg/kg [12]. Despite STE being 250−300 times sweeter than sucrose, it has several nutritional and medical activities such as anti-hyperglycaemic [13], anti-hypertensive [14], and anti-tumor activities [15]. Furthermore, several studies have shown that STE exerts anti-inflammatory and antioxidant effects both in vivo and in vitro [11,14,16]. In rats, STE could prevent liver inflammation through antioxidant activity by activating Nrf2 and anti-inflammatory activity by suppressing NF-κB signaling [11]. In a human colonic cell line, steviol (a derivative of STE) suppressed the IL-8 release induced by TNF-α, and reduced the protein expression of NF-κB [17]. STE could also attenuate the LPS-induced synthesis of pro-inflammatory cytokines by regulating IκBα/NF-κB signaling pathway [16]. In mice, STE promoted macrophage function and increased humoral immune response [18]. STE treatment enhanced antioxidant defense in both the adipose tissue and the vascular wall of obese insulin-resistant mice [19]. In addition, a recent study has indicated that dietary STE supplementation significantly increases serum IgG and IgA levels, and tends to increase the concentration of *Lactobacillus* in the cecal digesta of broilers [20]. However, most of the studies in chickens were mainly focused on the effects of dietary STE supplementation on the growth performance and the metabolism of chickens [12,21]. Whether STE has a regulatory function on the inflammation and oxidative stress of chicken intestinal mucosae still remains unclear.

Based on the findings above, we hypothesize that dietary STE supplementation can alleviate intestinal mucosal damage in broilers. The present study was designed to test this hypothesis using an LPS-induced intestinal mucosal damage model, and to further investigate whether STE exerts anti-inflammatory and antioxidant effects on the intestinal mucosae of LPS-challenged broilers.

## 2. Materials and Methods

### 2.1. Animals and Treatment

The animal experiments were performed in accordance with the Animal Care and Use Committee of Nanjing Agricultural University, Nanjing, China (PZ2019064). A total of 192 one-day-old male Ross 308 broiler chicks with similar hatching weights were purchased from a local commercial hatchery. Broilers were randomly allocated to four treatments. Each treatment contained six replicates (cages) of eight broilers per replicate in each group. The present experiment lasted for 21 d (from 1 to 21 d of age). The basal diet used in this study was according to National Research Council (1994) (Table 1). The four experimental treatments were as follows: (1) non-challenged broilers fed a basal diet (CON); (2) non-challenged broilers fed a basal diet supplemented with 250 mg/kg stevioside (STE); (3) LPS-challenged broilers fed a basal diet (LPS); (4) LPS-challenged broilers fed a basal diet supplemented with 250 mg/kg stevioside (LPS + STE). Stevioside used in this study were purchased from Macklin Inc (Shanghai, China) with a purity of more than 98%. The supplemental stevioside level was optimized according to previous studies [11,14,19]. All broilers were housed in four-level cages in a temperature- and light-controlled room with continuous light in Nanjing Agricultural University. All broilers had ad libitum access to mash feed and water. The temperature of the room was maintained at 32 to 34 °C for a week, and it was then gradually decreased by 1 °C every 2 d until a final temperature of 26 °C was achieved. Furthermore, all broilers were inoculated with a Newcastle disease vaccine on 7 d and with an inactivated infectious bursal disease vaccine on 14 d. The experiment consisted of a 2 × 2 factorial design. The main factors were (1) Lipopolysaccharide (LPS)-challenge, injection with LPS or saline, and (2) diet, basal diet with 0 or 250 mg/kg stevioside. LPS from *Escherichia coli* (L2880, Sigma Aldrich Inc., St. Louis, MO, USA) was dissolved in 0.9% sterile saline solution. At 7:00 am of 17, 19, and 21 d, LPS-challenged groups received an intraperitoneal injection of LPS solution at a dose of 1 mg/kg, whereas the CON and STE groups received a sterile saline injection. The dosage and injection of LPS were referred to as available findings [2,22]. At 17 d and 21 d, all broilers were weighed to calculate average daily gain (ADG). The feed consumption by the broilers in a replicate (cage) was recorded to calculate average daily feed intake (ADFI). The spilled feed was carefully collected and weighed in order to correct the final data of ADFI. Feed conversion rate (FCR) was defined as ADFI: ADG.

Provided the following % per kilogram in completed diet: vitamin A, 12,500 IU; vitamin D3, 2500 IU; vitamin E, 80 mg; vitamin K, 2.65 mg; vitamin B1, 2 mg; vitamin B2, 6 mg; nicotinic acid, 50 mg; pantothenic acid, 20 mg; vitamin B6, 4 mg; folic acid, 1.25 mg; vitamin B12, 0.025 mg; biotin, 0.0325 mg; folic acid, 1.25 mg; pantothenic acid, 12 mg; niacin, 50 mg; Fe, 80 mg; Zn, 75 mg; Mn, 100 mg; Cu, 8 mg; I, 0.35 mg; Co, 0.2 mg; and Se, 0.15 mg.

### 2.2. Sample Collection

Three hours after injection of LPS at 21 days of age, one broiler in each replicate (six broilers per treatment) with bodyweight close to the average body weight in the respective cage were selected. Blood samples were collected from the wing vein. The serum was then separated after centrifugation at 3000 g for 15 min at 4 °C, and it was stored at −20 °C for further analysis. After euthanasia by CO2 asphyxiation, the jejunum (anterior of Meckel’s diverticulum) and ileum (posterior to Meckel’s diverticulum) were then gingerly separated. Sections of approximately 2 cm in length were cut off from the middle of each jejunum and ileum. The jejunal and ileal sections were promptly fixed in 4% paraformaldehyde for histological analyses. The jejunal and ileal mucosae were gently scraped by a glass microscope slide from the rest of the jejunum and ileum. The mucosae were stored at −80 °C for the analysis of the oxidative status, gene expression, and protein expression.

### 2.3. Measurement of Serum Diamine Oxidase Activity

Diamine oxidase (DAO) activity in the serum was determined using a commercial reagent kit (Jin Yibai Biological Technology, Nanjing, China) according to the manufacturer’s instructions. The intra-assay coefficient of variation (CV) was < 9%, and the inter-assay coefficient of variation was < 10%.

### 2.4. Intestinal Morphology Analysis

After fixation in 4% paraformaldehyde for 24 h, the jejuna and ilea were soaked through a graded series of ethanol and xylene, embedded in paraffin, and sectioned at 5 μm with a Lecia RM2235 microtome (Leica Biosystems Inc., Buffalo Grove, USA). The sections were deparaffinized with xylene and rehydrated through graded dilutions of ethanol, and stained with hematoxylin and eosin. The images of jejuna and ilea were acquired using an Olympus simon-01 microscope (Olympus Optical Co., Ltd., Beijing, China). The values of villus height (VH) and crypt depth (CD) were measured 5 times from different villus and crypts per section from each broiler using the Image-Pro Plus software 6.0 (Media Cybernetics, Inc., Washington, USA). VCR was defined as villus height-to-crypt depth ratio.

### 2.5. Assessment of the Oxidative Status

The amount of 0.2 g frozen mucosa was precisely weighed and homogenized in 2 mL of ice-cold saline. After being centrifuged at 12,000× *g* for 10 min at 4 °C, the supernatants were collected to measure the oxidative status. The protein content of the supernatants was measured with a BCA Protein Assay Kit (P0010, Beyotime Biotechnology, Shanghai, China). We assessed malondialdehyde (MDA) content, total antioxidant capacity (T-AOC), catalase (CAT) activity, superoxide dismutase (SOD) activity, and glutathione peroxidase (GSH-Px) activity in the jejunal and ileal mucosae using commercial reagent kits (S0131, S0121, S0051, S0101 and S0056, Beyotime Biotechnology, Shanghai, China). All experimental procedures were performed according to the manufacturer’s instructions. All results were normalized to protein concentration in each sample.

### 2.6. TUNEL Assay

Intestinal apoptosis was determined using a terminal deoxynucleotidyl transferase-mediated deoxyuridine triphosphate nick-end labeling (TUNEL) assay with a TUNEL BrightGreen Apoptosis Detection Kit (A112, Vazyme Biotech, Nanjing, China) according to the manufacturer’s instructions. First, the paraffin sections of jejuna and ilea were deparaffinized, rehydrated, and then incubated with Proteinase K (20 μg/mL) at room temperature for 20 min. Second, the sections were incubated with the TdT enzyme buffer containing double-distilled H_2_O, Equilibration Buffer, BrightGreen Labeling Mix and Recombinant TdT Enzyme at 37 °C for 60 min in the dark. Finally, the sections were stained with 4′,6-diamidino-2-phenylindole staining solution (C1005, Beyotime Biotechnology, Shanghai, China) for 5 min in the dark. The negative control was performed as above, but without incubation of the TdT enzyme buffer to ensure that no non-specific reaction appeared in the experiment. The images were acquired through an LSM 700 confocal laser scanning microscope (Carl Zeiss, Oberkochen, Germany). The numbers of apoptotic cells (green color) and total cells (blue color) were counted using the Image-Pro Plus software 6.0 (Media Cybernetics, Inc., Washington, USA). The ratio of apoptotic cells to total cells represents the apoptotic index.

### 2.7. Western Blot

The jejunal and ileal mucosae were homogenized in Radio Immunoprecipitation Assay (RIPA) buffer containing phenylmethylsulfonyl fluoride (PMSF) to extract the proteins. Equal amounts of proteins (40 µg) were electrophoresed in 10% (*w*/*v*) SDS-PAGE, and then transferred on to polyvinylidene difluoride (PVDF) membranes (Millipore, Bedford, MA, USA). The membranes were blocked with TBST buffer containing 5% bovine serum albumin (BSA) for 2 h at room temperature and were incubated overnight at 4 °C with primary antibodies. The primary antibodies were NF-κB (1:1000, ab16502, Abcam, Cambridge, UK), p-NF-κB (1:1000, #3033, Cell Signaling Technology, MA, USA), IκBα (1:500, 10268-1-AP, Proteintech, Wuhan, China) and p-IκBα (1:1000, MA5-15087, Invitrogen, IL, USA), PCNA (1:1000, ab29, Abcam, Cambridge, UK) and β-actin (1:5000, 66009, Proteintech Group, Inc., IL, USA). After washing the membranes with TBST buffer for three times, the membranes were incubated with secondary antibody (1:3000, AS003, ABclonal Biotechnology Co., Ltd., Wuhan, China) for 60 min at room temperature. Finally, the blots were developed using an enhanced chemiluminescence (ECL) kit (Thermo Scientific, Wilmington, USA), and were visualized using a Luminescent Image Analyzer LAS-4000 (Fuji Film, Tokyo, Japan). The blots were normalized to β-actin. The intensities of the immunoreactive bands were quantified by ImageJ to estimate the protein expressions.

### 2.8. Total RNA Extraction and mRNA Quantification

The total RNA of intestinal mucosae was extracted using the RNAiso Plus (9109, Takara Bio Inc., Dalian, China). The concentration and quality of total RNA were identified by an ND-2000 microspectrophotometer (Thermo Scientific, Wilmington, USA.). The RNA samples with the 260/280 ratios of 1.8−2.0 and the 260/230 ratios of 2.0−2.2 were chosen for further reactions. Afterward, the RNA was reverse-transcribed into complementary DNA using a PrimeScript RT reagent Kit with gDNA Eraser (RR047A, Takara Bio Inc., Dalian, China). The gDNA Eraser was added to remove the DNA, and a total of 1 µg of RNA was reverse-transcribed to complementary DNA. Complementary DNA was diluted 10× before real-time PCR. Real-time PCR was performed using the TB Green Premix Ex Taq (RR420A, Takara Bio Inc., Dalian, China) on the QuantStudio 5 Real-Time PCR System (Thermo Scientific, Wilmington, USA). The β-actin gene was selected to be the housekeeping gene to normalize the expression of the other target genes. The primers were synthesized by Sangon Biotech (Sangon Biotech Co., Ltd., Shanghai, China), and the primer sequences for them were shown in Table 2. The reaction mixture of 20 μL included 10 μL of TB Green Premix Ex Taq, 0.4 μL of ROX Reference Dye II, 2 μL of cDNA template, 0.4 μL of each primer (10 μM) and 6.8 μL of double-distilled H_2_O. All genes were assayed three times. The reaction program was as follows: 95 °C for 30 s, 40 cycles of 95 °C for 5 s followed by 60 °C for 30 s. The melting curve was used to verify the amplification of a single product. Relative gene expression levels were analyzed by the 2^−ΔΔCt^ method after normalization against β-actin.

*TLR4*, toll-like receptor 4; *MyD88*, myeloid differentiation factor 88; *NF-κB*, nuclear factor kappaB; *TNF-α*, tumor necrosis factor-alpha; *IFN-γ*, interferon-γ; *IL-1β*, interleukin 1 beta; *IL-6*, interleukin 6; *Nrf2*, nuclear factor-erythroid 2-related factor 2; *HO-1*, heme oxygenase-1; *SOD1*, superoxide dismutase-1; *SOD2*, superoxide dismutase-2; *CAT*, catalase; *GPX1*, glutathione peroxidase 1; *CLDN1*, claudin-1; *CLDN2*, claudin-2; *OCLN*, occludin; *ZO-1*, zonula occludens-1; *PCNA*, proliferating cell nuclear antigen.

### 2.9. Statistical Analysis

Data were statistically analyzed by two-way ANOVA in a 2 × 2 factorial arrangement with LPS challenge and stevioside supplementation as the main effects and their interaction, using SPSS software (SPSS 20.0, SPSS, Chicago, USA). The Shapiro-Wilk test was used to assess the normality distribution of the data. When the interaction was observed to be significant, Tukey’s multiple range tests were applied to examine the statistical differences among different treatments. Differences were considered to be statistically significant at *p* < 0.05, and 0.05 < *p* < 0.10 was considered to be a trend towards significance.

## 3. Results

### 3.1. Growth Performance

The interaction between STE supplementation and LPS challenge notably affected average daily feed intake (*p* < 0.05). The LPS-challenged group had a significantly lower (*p* < 0.05) average daily feed intake than the other groups (Table 3). LPS challenge also reduced (*p* < 0.05) average daily gain of broiler chickens. No differences (*p* > 0.05) were observed in the feed conversion rate among any groups.

### 3.2. Intestinal Permeability and Morphology

**Figure 1:**
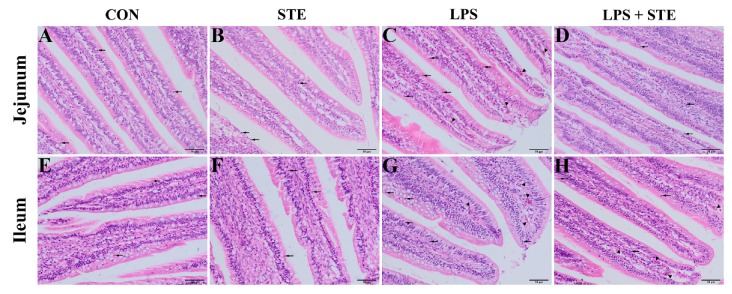
Representative images of jejunal (**A**–**D**) and ileal (**E**–**H**) morphology of D21 broilers. Triangle and arrow represent hyperemia and neutrophil infiltrations, respectively. CON, non-challenged broilers fed a basal diet; STE, non-challenged broilers fed a basal diet supplemented with 250 mg/kg stevioside; LPS, LPS-challenged broilers fed a basal diet; LPS + STE, LPS-challenged broilers fed a basal diet supplemented with 250 mg/kg stevioside. Scale bar = 50 μm.

We performed H&E staining to observe the effects of LPS challenge and STE supplementation on the intestinal morphology of broilers. Almost no damages were found in either the CON group (Figure 1A,E) or the STE group (Figure 1B,F). LPS injection could cause obvious hyperemia (Figure 1C,G, triangles) and neutrophil infiltration (Figure 1C,G, arrows) in jejunal and ileal mucosae. The LPS + STE group (Figure 1D,H) had reduced hyperemia and neutrophil infiltration compared with the LPS group.

The interaction between STE supplementation and LPS challenge notably affected serum DAO level (*p* < 0.05). The LPS-challenged group had a higher (*p* < 0.05) serum DAO level than the non-challenged groups (Table 4). STE supplementation reduced the serum DAO level of LPS-challenged broilers (*p* < 0.05).

As shown in Table 4, significant interactions between STE supplementation and LPS challenge were observed for CD and VCR in both jejunum and ileum (*p* < 0.05). LPS challenge significantly decreased VH and VCR, but increased CD in both jejunum and ileum (*p* < 0.05). Dietary supplementation of STE significantly reduced CD and increased VH and VCR in both jejunum and ileum (*p* < 0.05).

### 3.3. Inflammatory Gene Expression

There was an LPS × STE interaction (*p* < 0.05) on the mRNA expression of *TLR4*, *MyD88*, *TNF-α,* and *IFN-γ* in the jejunal mucosa of broilers (Table 5). The LPS challenge resulted in enhanced mRNA expression of *TLR4*, *MyD88*, *TNF-α,* and *IFN-γ* in the jejunal mucosa compared with the CON group (*p* < 0.05). The enhanced mRNA expressions of these aforementioned genes were reversed by STE supplementation (*p* < 0.05). Moreover, the LPS challenge significantly increased the mRNA expressions of *NF-κB*, *IL-1β,* and *IL-6* in the jejunal mucosa (*p* < 0.05). LPS challenge also enhanced the mRNA expressions of *NF-κB*, *IFN-γ,* and *IL-1β* in the ileal mucosa (*p* < 0.05), and tended to up-regulate the expressions of jejunal *TNF-α* (*p* = 0.071)*,* ileal *TLR4* (*p* = 0.053), and ileal *MyD88* (*p* = 0.099). Dietary STE supplementation significantly reduced the mRNA expression of *NF-κB* in the ileal mucosa (*p* < 0.05), and tended to down-regulate the expressions of jejunal *IL-1β* (*p* = 0.052) and ileal *IFN-γ* (*p* = 0.067). In addition, LPS challenge had no effect on the expressions of ileal *TNF-α* and *IL-6* (*p* > 0.05). STE supplementation did not alter mRNA abundances of *TLR4*, *MyD88*, *NF-κB*, *TNF-α*, *IL-1β,* and *IL-6* in the ileal mucosa, and *IL-6* mRNA abundance in the jejunal mucosa (*p* > 0.05).

### 3.4. Protein Expression of NF-κB, p-NF-κB, IκBα and p-IκBα

**Figure 2:**
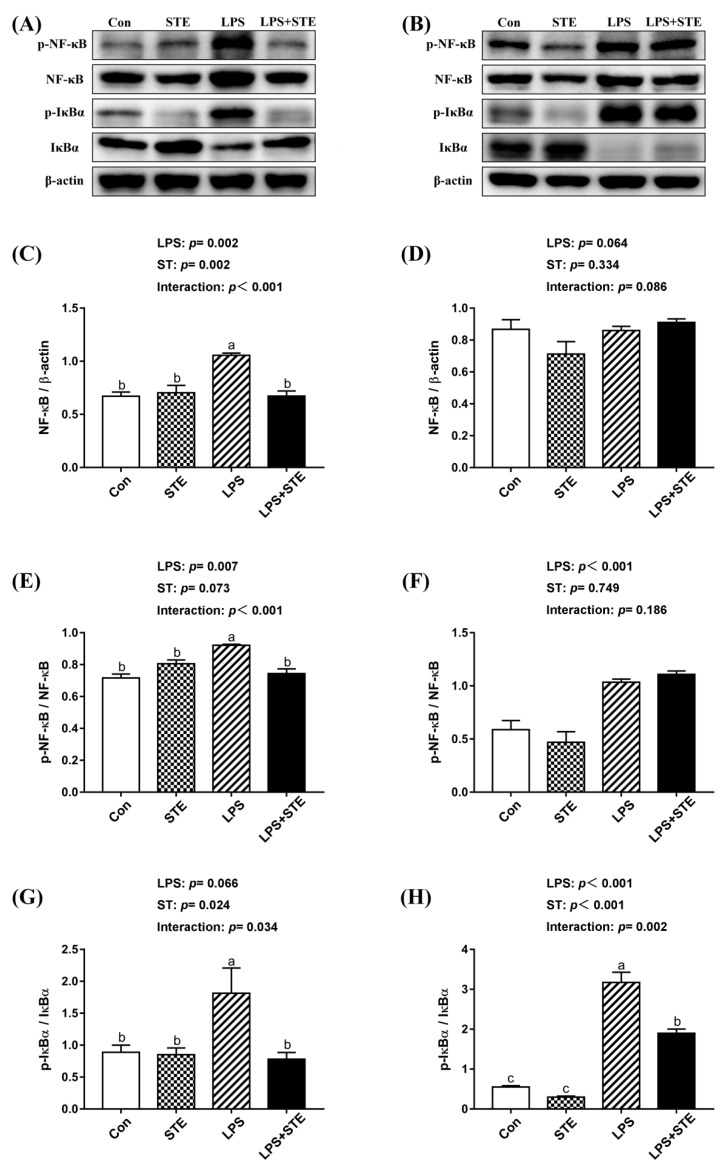
Effects of stevioside supplementation on the protein expression of NF-κB, p-NF-κB, IκBα and p-IκBα. (**A**) Western blot analysis of NF-κB, p-NF-κB, IκBα and p-IκBα in the jejunal mucosae. (**B**) Western blot analysis of NF-κB, p-NF-κB, IκBα and p-IκBα in the ileal mucosae. (**C**) Statistical analysis of NF-κB/ β-actin in the jejunal mucosae. (**D**) Statistical analysis of NF-κB/ β-actin in the ileal mucosae. (**E**) Statistical analysis of p-NF-κB/ NF-κB in the jejunal mucosae. (**F**) Statistical analysis of p-NF-κB/ NF-κB in the ileal mucosae. (**G**) Statistical analysis of p-IκBα/ IκBα in the jejunal mucosae. (**H**) Statistical analysis of p-IκBα/ IκBα in the ileal mucosae. CON, non-challenged broilers fed a basal diet; STE, non-challenged broilers fed a basal diet supplemented with 250 mg/kg stevioside; LPS, LPS-challenged broilers fed a basal diet; LPS + STE, LPS-challenged broilers fed a basal diet supplemented with 250 mg/kg stevioside. Data are presented as mean value ± SEM (*n* = 6).^a,b,c^ Means with different letters are significantly different (*p* < 0.05).

We observed notable interaction (*p* < 0.05) between STE supplementation and LPS challenge on the protein expression of ileal p-IκBα, jejunal NF-κB, p-NF-κB, and p-IκBα in the intestinal mucosae of broilers (Figure 2). LPS-challenged group exhibited extremely higher (*p* < 0.05) expression in jejunal NF-κB, p-NF-κB, and p-IκBα. But STE supplementation significantly reduced (*p* < 0.05) the expression of these proteins. The LPS challenge also increased (*p* < 0.05) the protein expression of p-IκBα in the ileal mucosa, while STE supplementation reduced it (*p* < 0.05). Moreover, LPS administration elevated (*p* < 0.05) the protein expression of p-NF-κB in the ileal mucosa, whereas STE supplementation had no protective effect on it (*p* > 0.05).

### 3.5. Tight Junction-Related Genes Expression

We observed a significant interaction (*p* < 0.05) between STE supplementation and LPS challenge on the mRNA expression of *CLDN2*, *OCLN,* and *ZO-1* in the jejunal mucosa of broilers (Table 6). The mRNA abundances of *CLDN2*, *OCLN,* and *ZO-1* in the jejunal mucosa decreased (*p* < 0.05) in the LPS group, while no significant differences were observed in the LPS + STE group compared with the CON group (*p* > 0.05). LPS challenge resulted in decreased (*p* < 0.05) expression of *OCLN*, whereas STE supplementation had no significant effect in ileal *OCLN* expression (*p* > 0.05). There was no effect of either LPS challenge or STE supplementation on *CLDN1*, *CLDN2,* and *ZO-1* expression in the ileal mucosa (*p* > 0.05).

### 3.6. Oxidative Status

As shown in Table 7, we observed significant interactions between STE supplementation and LPS challenge in T-AOC (jejunal mucosa), MDA content (ileal mucosa), and SOD activity (ileal mucosa) of broilers (*p* < 0.05). Compared with broilers in non-challenged groups, LPS challenge markedly increased (*p* < 0.05) MDA content (jejunal and ileal mucosa) but decreased (*p* < 0.05) T-AOC (ileal mucosa), CAT activity (jejunal and ileal mucosa), and GSH-Px activity (jejunal and ileal mucosa). Broilers receiving STE administration had lower (*p* < 0.05) MDA content (jejunal and ileal mucosa) but higher (*p* < 0.05) T-AOC (jejunal mucosa), SOD activity (ileal mucosa), CAT activity (jejunal mucosa), and GSH-Px activity (jejunal mucosa). STE supplementation also tended to increase SOD activity in jejunal mucosa (*p* = 0.092). In addition, the LPS challenge had no effect on jejunal T-AOC, jejunal SOD activity, and ileal SOD activity (*p* > 0.05). STE supplementation did not alter T-AOC, CAT and GSH-Px activity in the ileal mucosa (*p* > 0.05).

### 3.7. Antioxidant Gene Expression

As shown in Table 8, there was an LPS × STE interaction for the mRNA expression of *SOD2* and *Nrf2* in the jejunal mucosa of broilers (*p* < 0.05). LPS challenge significantly decreased the expression of *SOD1*, *CAT,* and *HO-1* in both jejunal and ileal mucosae (*p* < 0.05). LPS-challenged broilers also showed lower expression of *SOD2*, *GPX1* and *Nrf2* in the jejunal mucosa (*p* < 0.05). In addition, supplementation with STE significantly increased the expression of *SOD1*, *CAT,* and *Nrf2* in both jejunal and ileal mucosa (*p* < 0.05). The expression of jejunal *HO-1* (*p* = 0.052) and ileal *GPX1* (*p* = 0.086) tended to be increased by STE supplementation. LPS challenge had no effect on the mRNA expressions of ileal *SOD2*, *GPX1,* and *Nrf2* (*p* > 0.05). There was no effect of STE supplementation on *SOD2* expression in the jejunal mucosa (*p* > 0.05).

### 3.8. Apoptotic Index by TUNEL Assay

**Figure 3:**
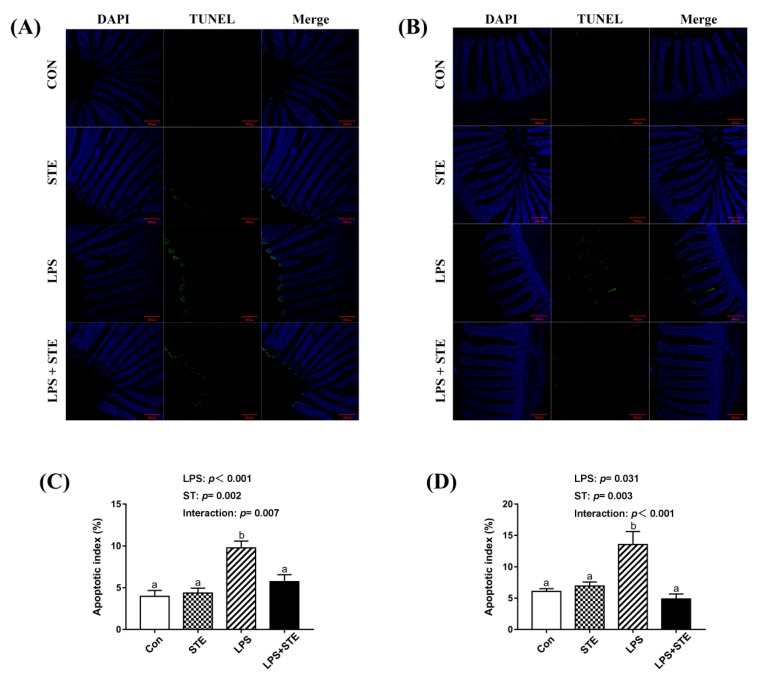
TUNEL assay of jejunal (**A**) and ileal (**B**) sections of LPS-challenged broilers by immunofluorescence. The blue color represents the total cells, and the green color represents the apoptosis cells in the jejunum and ileum. (**C**) Statistical analysis of apoptotic index in the jejunum. (**D**) Statistical analysis of apoptotic index in the ileum. CON, non-challenged broilers fed a basal diet; STE, non-challenged broilers fed a basal diet supplemented with 250 mg/kg stevioside; LPS, LPS-challenged broilers fed a basal diet; LPS + STE, LPS-challenged broilers fed a basal diet supplemented with 250 mg/kg stevioside. Data are presented as mean value ± SEM (*n* = 6). ^a,b^ Means with different letters are significantly different (*p* < 0.05). Scale bar = 200 μm.

The apoptotic cells were primarily distributed to the apical region of the jejunal and ileal villus (Figure 3A,B). A significant interaction (*p* < 0.05) between STE supplementation and LPS challenge was observed for the apoptotic index in both jejunal and ileal mucosae (Figure 3C,D). LPS-challenged broilers exhibited a greater percentage in both jejunal and ileal mucosae than the other groups (*p* < 0.05). In contrast, the LPS + STE group had a lower apoptotic index in both jejunal and ileal mucosae than the LPS group (*p* < 0.05).

### 3.9. Protein and mRNA Expression of PCNA

**Figure 4:**
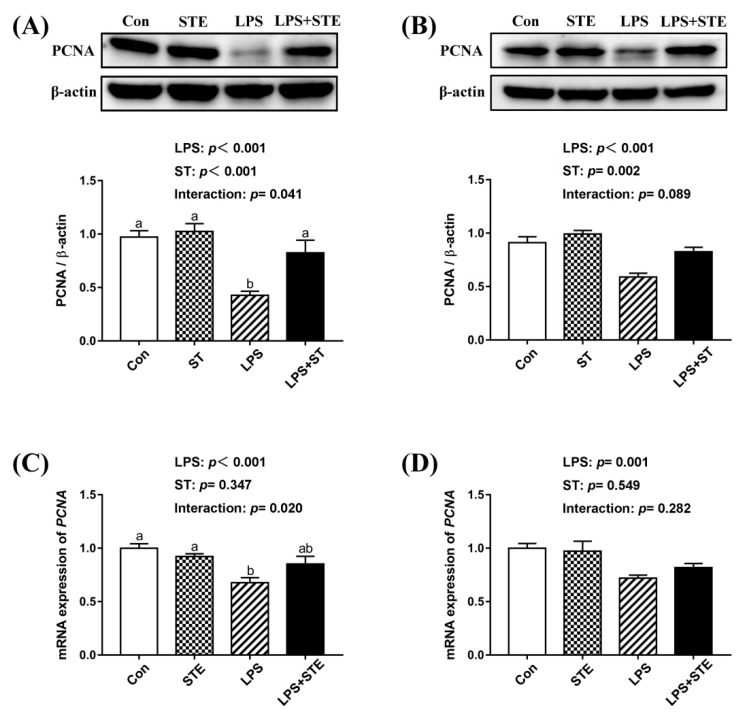
Effects of stevioside supplementation on the intestinal mucosal proliferation. (**A**) Western blot analysis of PCNA protein in the jejunal mucosae. (**B**) Western blot analysis of PCNA protein in the ileal mucosae. (**C)** The mRNA expression level of *PCNA* in the jejunal mucosae. (**D**) The mRNA expression level of *PCNA* in the ileal mucosae. CON, non-challenged broilers fed a basal diet; STE, non-challenged broilers fed a basal diet supplemented with 250 mg/kg stevioside; LPS, LPS-challenged broilers fed a basal diet; LPS + STE, LPS-challenged broilers fed a basal diet supplemented with 250 mg/kg stevioside. Data are presented as mean value ± SEM (*n* = 6). ^a,b^ Means with different letters are significantly different (*p* < 0.05).

We observed a significant interaction (*p* < 0.05) between STE supplementation and LPS challenge on the protein and mRNA expression of PCNA in the jejunal mucosa (Figure 4). LPS challenge significantly decreased the protein and mRNA expression of *PCNA* in both jejunal and ileal mucosae (*p* < 0.05). Dietary STE supplementation increased the protein expression of PCNA in both jejunal and ileal mucosae (*p* < 0.05). There was no effect of STE supplementation on the mRNA expression of *PCNA* in the ileal mucosa (*p* > 0.05).

## 4. Discussion

Intestinal health is essential for the growth of animals. Many pathogenic factors, including oxidative stress and pathogenic bacterial challenges, especially APEC, can damage the intestinal barrier and cause intestinal inflammation [7,23,24]. Ample pieces of evidence have proven that LPS is able to disrupt the intestinal barrier of broilers by bacterial challenge, which could cause a reduction in feed intake [25,26,27]. In addition, LPS challenge induced obvious hyperemia and more neutrophil infiltration. Mucosal hyperemia is the main change in chickens that responded with intestinal lesions [28]. Neutrophil infiltration is a prominent feature of inflammatory reactions. In keeping with our findings, Li et al. [8] found that a greater amount of neutrophils could be observed in the duodenum of LPS-challenged broilers. Additionally, several studies have demonstrated that LPS is able to induce gut inflammatory responses in broilers by increasing pro-inflammatory cytokine levels [2,7,8]. The release of pro-inflammatory cytokines requires the activation of TLR4 [29]. TLR4 serves as a receptor of LPS, and LPS can combine with it to activate a TLR4/MyD88/NF-κB signaling pathway in the intestine epithelial cell [30]. In this study, the transcription level of pro-inflammatory genes and the protein expression of p-NF-κB significantly increased in the intestinal mucosae of LPS-challenged broilers. NF-κB plays a crucial role in regulating inflammation and cell death, and it is inhibited by IκB. The phosphorylation of serine residues on IκBα could degrade IκBα, which, in turn, induces phosphorylation and nuclear translocation of NF-κB [31]. Phosphorylated NF-κB activated by TLR4 or TNF-α signaling could subsequently lead to the synthesis and release of pro-inflammatory cytokines [32]. However, dietary STE supplementation was capable of alleviating the phosphorylation of IκBα and NF-κB, which resulted in normalizing the transcription level of pro-inflammatory cytokines in the intestinal mucosae of broilers. Similarly, Casas-Grajales et al. [11] reported that STE could prevent liver inflammation in rats by reducing the expression level of NF-κB, TNF-α and pro-inflammatory cytokines (IL-17a, IL-1β, and IL-6). Boonkaewwan et al. [16] demonstrated that STE inhibited LPS-induced synthesis of pro-inflammatory cytokines in human colonic epithelial cells. Alavala et al. [33] indicated that STE was beneficial for treating ulcerative colitis by inhibiting NF-κB and MAPK pathways. Diterpenoid moiety in the structure of STE may contribute to the anti-inflammatory activity in STE [34,35]. Thus, our results suggest that dietary STE supplementation could attenuate LPS-induced inflammation by attenuating the TLR4/NF-κB signaling pathway in the intestine of broilers.

Intestinal integrity is a key factor for preventing pathogenic microorganism invasion in broiler chickens. We found that the LPS challenge severely impaired the intestinal morphology in broilers, as indicated by decreased VH and VCR in both jejunum and ileum. The VH is related to the ability to absorb nutrients in the intestine. A high VCR is widely believed to be a good index of mucosal turnover, and it is associated with the strong ability of digestion and absorption [7,36]. LPS-induced structural damage of the intestinal mucosal barrier was reversed with dietary STE supplementation. It has been reported that natural extract with anti-inflammatory effect can restore the damaged intestinal morphology in LPS-challenged broilers [37,38]. The down-regulation of TLR4/NF-κB signaling and reduced mRNA expression of pro-inflammatory cytokines might be one of the reasons that intestinal integrity improved in the LPS + STE group. Our results indicate that dietary STE supplementation can ameliorate the impaired intestinal integrity in LPS-challenged broilers.

DAO is an intracellular enzyme produced by the intestinal epithelium and mainly exists in the intestinal mucosae [39]. Once the intestinal mucosal barrier is impaired, DAO is released into the systemic blood. Thus, serum DAO activity is a marker for intestinal permeability. Our result was accorded with the previous finding that LPS impaired the intestinal permeability [7,40]. Furthermore, intestinal mucosal barrier functions are mainly regulated by tight junctions [41]. The reduced expression level of tight junctions caused by LPS challenge could affect intestinal permeability and cause pathological states [42]. This result was consistent with increased serum DAO activity. Additionally, previous studies have demonstrated that increased IFN-γ and IL-1β would damage the tight junctions and disrupt intestinal permeability [43,44]. However, dietary STE supplementation repaired the intestinal permeability. The exact mechanisms for this effect of STE require further investigation. Our data reveal that STE administration could restore the disrupted intestinal permeability of LPS-challenged broilers.

The LPS challenge can not only induce inflammation but also induce oxidative stress [45,46]. Increased protein expression of p-IκBα and p-NF-κB also reflected that strong oxidative stress was induced by LPS challenge [47]. Oxidative stress is a pivotal factor for disrupted mucosal barrier function [48]. MDA is the chief oxidative degradation product, and it reflects the level of lipid peroxidation [49]. The antioxidant defense system mainly consists of T-AOC, CAT, SOD, and GSH-Px. T-AOC is one of the most important indices reflecting the total antioxidant capacity as a single measure [50]. CAT, SOD, and GSH-Px act as free radical scavengers to decompose H_2_O_2_. In this study, LPS induced lipid peroxidation damage in the intestinal mucosae. Our result of decreased MDA content by STE supplementation was consistent with the previous finding that STE significantly reversed the lipid peroxidation in the liver of the LPS-challenged rat [49]. Increasing evidence has shown that STE exerts potent antioxidant properties in vivo and in vitro [11,49,51,52]. In agreement with these findings, we also demonstrated that STE markedly increased the total antioxidant capacity, and reduced oxidative stress in the intestinal mucosae of LPS-challenged broilers. The transcription level of *SOD1*, *SOD2*, *CAT,* and *GPX1* coincided with the data of antioxidant enzyme activity. Moreover, HO-1 is a major antioxidant enzyme, and its transcription is primarily under the control of Nrf2 [53]. It is well established that the Nrf2-HO-1 pathway is critical for redox balance. Our result of increased mRNA expression of *Nrf2* and *HO-1* suggested that STE could exert antioxidant effect via activation of Nrf2-HO-1 signaling. Similarly, a previous study has indicated that rebaudioside A, one of the steviol glycosides besides STE, considerably induces the Nrf2 cascade [54]. Casas-Grajales et al. [11] have also demonstrated that STE is able to prevent thioacetamide-induced liver damage through up-regulating Nrf2, thus preserving the normal redox status in rats. Based on these results, we hypothesize that STE exerts its antioxidant potential by inducing the Nrf2 cascade and enhancing the antioxidant enzyme activity. However, further functional studies are required to validate this hypothesis.

The balance of cell apoptosis and proliferation are important for maintaining the turnover of the intestinal mucosal epithelium [55]. Inflammation and oxidative stress can induce excessive apoptosis and declined proliferation, which could lead to intestinal barrier dysfunction [56,57]. In the present study, we observed apoptosis was promoted in the intestinal mucosae of LPS-challenged broilers using a TUNEL assay. Supportively, LPS increased pro-apoptotic Bax and Caspase-3 expression and decreased anti-apoptotic Bcl-2 expression in the duodenal mucosa of broilers [8]. However, enhanced apoptosis induced by LPS injection was alleviated by STE administration. Emerging evidence has indicated that STE has an anti-apoptotic effect on rodents [49,58,59]. Our results of decreased *NF-κB* expression level and reduced apoptosis were in agreement with a previous finding that STE could suppress apoptosis via a mechanism involving ERK1/2, STAT3 and NF-κB suppression [59]. The reduced apoptosis of LPS-challenged broilers might also be attributable to the anti-inflammatory and antioxidant effects of STE supplementation. On the other hand, both protein and transcription levels of PCNA (an indicator for cell proliferation) in the intestinal mucosae significantly declined after LPS injection. This decline of PCNA was also counteracted by STE supplementation. Similarly, a previous study showed that STE substantially increased cell proliferation in the LPS-stimulated T- and B- lymphocytes ex vivo [18]. STE administration could enhance macrophage function and resulted in modulating the T and B cell proliferation [18]. This increases the possibility that STE could modulate cell proliferation through immunomodulatory activity. Hence, our results suggest that STE can inhibit apoptosis and promote cell proliferation in the intestinal mucosae of LPS-challenged broilers.

Interestingly, our data showed that dietary STE supplementation had better effects on LPS-challenged jejunum than ileum on p-NF-κB expression, the transcription level of tight junction, and pro-inflammatory cytokines. A previous study has reported that the LPS challenge could cause numerous changes in microflora [60]. Since ileum has more abundant microflora, LPS might induce more irreversible damage to the ileal mucosa. Furthermore, stevioside is hydrolyzed to steviol by the intestinal microflora [61]. It has been shown that stevioside could suppress the activation of NF-κB and the release of TNF-α and IL-1β induced by LPS in THP-1 cells, whereas steviol did not have the same effect [62]. We presume that after long detention time in the intestine, more part of stevioside is hydrolyzed to steviol in the ileum. Stevioside might exert better anti-inflammatory and antioxidant effects on the intestinal mucosae than steviol, which could explain the differences in the effects of stevioside in different intestinal segments. This speculation requires further investigation.

## 5. Conclusions

In conclusion, dietary stevioside supplementation could alleviate LPS-induced chicken intestinal mucosal damage by ameliorating inflammation and improving the antioxidant status of intestinal mucosae. Our results have shown that stevioside has anti-inflammatory and antioxidant effects. Therefore, stevioside can be used as a feed supplement in preventing intestinal inflammatory disease in broiler chickens.

## Figures and Tables

**Table 1:** Ingredient composition and calculation of ingredients of the basal diet for broiler.

Items	1 to 21 d
Ingredient (%)	
Corn	53.28
Soybean meal	38.57
Soybean oil	3.70
Dicalcium phosphate	1.98
Mineral premix	0.50
Vitamin premix	0.10
Limestone	1.05
Choline chloride (50%)	0.30
Salt	0.35
Methionine	0.17
Total	100
Calculation of nutrients	
Metabolizable energy, kcal/kg	2.953
Crude protein, %	21.57
Lysine, %	1.15
Methionine, %	0.49
Calcium, %	1.05
Available phosphorus, %	0.45

**Table 2:** Primer sequences used for RT-qPCR in this study.

Gene	Primer Sequence (5′ → 3′)	Amplicon Size (bp)	GeneBank Accession Number
*TLR4*	forward: AGGCACCTGAGCTTTTCCTC	96	NM_001030693.1
	reverse: TACCAACGTGAGGTTGAGCC		
*MyD88*	forward: TGATGCCTTCATCTGCTACTG	174	NM_001030962.4
	reverse: TCCCTCCGACACCTTCTTTCTA		
*NK-κB*	forward: GTGTGAAGAAACGGGAACTG	203	NM_205129.1
	reverse: GGCACGGTTGTCATAGATGG		
*TNF-α*	forward: GAGCGTTGACTTGGCTGTC	64	XM_015294120.2
	reverse: AAGCAACAACCAGCTATGCAC		
*IFN-γ*	forward: AACAACCTTCCTGATGGCGT	106	NM_205149.1
	reverse: TGAAGAGTTCATTCGCGGCT		
*IL-1β*	forward: CCGAGGAGCAGGGACTTT	132	XM_015297469.1
	reverse: GGACTGTGAGCGGGTGTAG		
*IL-6*	forward: TTTATGGAGAAGACCGTGAG	105	NM_204628.1
	reverse: GTGGCAGATTGGTAACAGAG		
*Nrf2*	forward: GATGTCACCCTGCCCTTAG	216	NM_205117.1
	reverse: CTGCCACCATGTTATTCC		
*HO-1*	forward: GCGGAGAACACACCCTTCAT	235	NM_205344.1
	reverse: GGATCTCTGCCCTCCAGTTG		
*SOD1*	forward: TTGTCTGATGGAGATCATGGCTTC	98	NM_205064.1
	reverse: TGCTTGCCTTCAGGATTAAAGTGAG		
*SOD2*	forward: CAGATAGCAGCCTGTGCAAATCA	86	NM_204211.1
	reverse: GCATGTTCCCATACATCGATTCC		
*CAT*	forward: GTTGGCGGTAGGAGTCTGGTCT	182	NM_001031215.2
	reverse: GTGGTCAAGGCATCTGGCTTCTG		
*GPX1*	forward: TCACCATGTTCGAGAAGTGC	124	NM_001277853.2
	reverse: ATGTACTGCGGGTTGGTCAT		
*CLDN1*	forward: CATACTCCTGGGTCTGGTTGGT	100	NM_001013611.2
	reverse: GACAGCCATCCGCATCTTCT		
*CLDN2*	forward: CTGCTCACCCTCATTGGA	140	NM_001277622.1
	reverse: AACTCACTCTTGGGCTTCTG		
*OCLN*	forward: ACGGCAGCACCTACCTCAA	123	NM_205128.1
	reverse: GGGCGAAGAAGCAGATGAG		
*ZO-1*	forward: CTTCAGGTGTTTCTCTTCCTCCTC	131	XM_015278975.2
	reverse: CTGTGGTTTCATGGCTGGATC		
*PCNA*	forward: GACAATGCGGATACGTTGGC	188	NM_204170.2
	reverse: TCACCAATGTGGCTGAGGTC		
*β-actin*	forward: TGTTACCAACACCCACACCC	110	NM_205518.1
	reverse: TCCTGAGTCAAGCGCCAAAA		

**Table 3:** Effects of stevioside supplementation on the growth performance of LPS- (lipopolysaccharide) challenged broilers.

	Treatments ^2^					*p* value		
Items ^1^	CON	STE	LPS	LPS + STE	SEM	LPS	STE	Interaction
17–21d								
ADFI (g/d)	85.29 ^a^	83.99 ^a^	72.07 ^b^	82.66 ^a^	3.56	0.009	0.081	0.029
ADG (g/d)	54.03	52.93	43.95	49.65	2.34	0.012	0.345	0.177
FCR	1.57	1.61	1.65	1.69	0.04	0.276	0.631	0.969

^1^ ADFI, average daily feed intake; ADG, average daily gain; FCR, feed conversion rate; ^2^ CON, non-challenged broilers fed a basal diet; STE, non-challenged broilers fed a basal diet supplemented with 250 mg/kg stevioside; LPS, LPS-challenged broilers fed a basal diet; LPS + STE, LPS-challenged broilers fed a basal diet supplemented with 250 mg/kg stevioside; SEM, standard error of mean; ^a,b^ Means (*n* = 6) with different letters within a row are significantly different (*p* < 0.05).

**Table 4:** Effects of stevioside supplementation on serum diamine oxidase activity and intestinal morphology of LPS-challenged broilers.

	Treatments ^2^					*p* value		
Items ^1^	CON	STE	LPS	LPS + STE	SEM	LPS	STE	Interaction
Serum								
DAO (U/L)	62.34 ^b^	63.51 ^b^	80.15 ^a^	66.96 ^b^	2.83	0.001	0.054	0.023
Jejunum								
Villus height (μm)	1118.18	1155.08	973.94	1088.91	24.98	<0.001	0.005	0.135
Crypt depth (μm)	135.34 ^b^	141.44 ^b^	150.87 ^a^	142.15 ^a,b^	2.33	0.002	0.590	0.004
VCR	8.27 ^a^	8.18 ^a^	6.47 ^b^	7.70 ^a^	0.23	<0.001	0.019	0.007
Ileum								
Villus height (μm)	778.28	798.76	622.92	712.08	20.09	<0.001	0.012	0.103
Crypt depth (μm)	109.36 ^b^	110.05 ^b^	136.89 ^a^	114.42 ^b^	2.96	<0.001	<0.001	<0.001
VCR	7.15 ^a^	7.31 ^a^	4.59 ^b^	6.42 ^a^	0.23	<0.001	<0.001	0.002

^1^ DAO, diamine oxidase; VCR, villus height-to-crypt depth ratio; ^2^ CON, non-challenged broilers fed a basal diet; STE, non-challenged broilers fed a basal diet supplemented with 250 mg/kg stevioside; LPS, LPS-challenged broilers fed a basal diet; LPS + STE, LPS-challenged broilers fed a basal diet supplemented with 250 mg/kg stevioside; SEM, standard error of mean; ^a,b^ Means (*n* = 6) with different letters within a row are significantly different (*p* < 0.05).

**Table 5:** Effects of stevioside supplementation on inflammatory gene expression in the intestinal mucosae of LPS-challenged broilers.

	Treatments ^2^					*p* value		
Items ^1^	CON	STE	LPS	LPS + STE	SEM	LPS	STE	Interaction
Jejunum								
*TLR4*	1.00 ^b^	0.98 ^b^	1.58 ^a^	0.99 ^b^	0.12	0.042	0.035	0.049
*MyD88*	1.00 ^b^	1.04 ^b^	1.52 ^a^	1.10 ^b^	0.05	<0.001	0.005	0.001
*NF-κB*	1.00	0.90	1.33	0.99	0.07	0.008	0.006	0.098
*TNF-α*	1.00 ^b^	0.99 ^b^	1.40 ^a^	0.97 ^b^	0.08	0.071	0.047	0.045
*IFN-γ*	1.00 ^c^	1.26 ^b,c^	4.17 ^a^	2.32 ^b^	0.29	<0.001	0.023	0.004
*IL-1β*	1.00	1.41	3.99	5.69	0.44	<0.001	0.052	0.216
*IL-6*	1.00	1.18	2.12	1.50	0.19	0.003	0.305	0.076
Ileum								
*TLR4*	1.00	1.06	0.95	0.72	0.08	0.053	0.365	0.142
*MyD88*	1.00	1.04	1.39	1.13	0.07	0.099	0.214	0.102
*NF-κB*	1.00	1.02	1.37	1.19	0.07	0.003	0.324	0.218
*TNF-α*	1.00	0.89	0.86	0.93	0.08	0.548	0.807	0.306
*IFN-γ*	1.00	0.81	1.75	1.32	0.13	<0.001	0.067	0.449
*IL-1β*	1.00	1.15	2.63	3.37	0.29	<0.001	0.233	0.429
*IL-6*	1.00	1.01	1.09	1.40	0.14	0.102	0.288	0.304

^1^*TLR4*, toll-like receptor 4; *MyD88*, myeloid differentiation factor 88; *NF-κB*, nuclear factor kappaB; *TNF-α*, tumor necrosis factor-alpha; *IFN-γ*, interferon-γ; *IL-1β*, interleukin 1 beta; *IL-6*, interleukin 6; ^2^ CON, non-challenged broilers fed a basal diet; STE, non-challenged broilers fed a basal diet supplemented with 250 mg/kg stevioside; LPS, LPS-challenged broilers fed a basal diet; LPS+STE, LPS-challenged broilers fed a basal diet supplemented with 250 mg/kg stevioside; SEM, standard error of mean; ^a,b^^,c^ Means (*n* = 6) with different letters within a row are significantly different (*p* < 0.05).

**Table 6:** Effects of stevioside supplementation on tight junction-related genes expression in the intestinal mucosae of LPS-challenged broilers.

	Treatments ^2^					*p* value		
Items ^1^	CON	STE	LPS	LPS + STE	SEM	LPS	STE	Interaction
Jejunum								
*CLDN1*	1.00	0.86	0.75	0.95	0.08	0.324	0.701	0.055
*CLDN2*	1.00 ^a^	0.76 ^a,b^	0.47 ^b^	0.68 ^a,b^	0.10	0.006	0.893	0.033
*OCLN*	1.00 ^a^	0.87 ^a,b^	0.70 ^b^	0.84 ^a,b^	0.06	0.023	0.751	0.030
*ZO-1*	1.00 ^a^	0.94 ^a^	0.67 ^b^	1.04 ^a^	0.06	0.094	0.028	0.004
Ileum								
*CLDN1*	1.00	1.02	0.96	1.06	0.12	0.996	0.644	0.772
*CLDN2*	1.00	0.72	0.79	0.73	0.15	0.515	0.297	0.477
*OCLN*	1.00	0.98	0.64	0.87	0.07	0.010	0.217	0.131
*ZO-1*	1.00	0.99	0.88	1.19	0.09	0.687	0.174	0.143

^1^*CLDN1*, claudin-1; *CLDN2*, claudin-2; *OCLN*, occludin; *ZO-1*, zonula occludens-1; ^2^ CON, non-challenged broilers fed a basal diet; STE, non-challenged broilers fed a basal diet supplemented with 250 mg/kg stevioside; LPS, LPS-challenged broilers fed a basal diet; LPS+STE, LPS-challenged broilers fed a basal diet supplemented with 250 mg/kg stevioside; SEM, standard error of mean; ^a,b^ Means (*n* = 6) with different letters within a row are significantly different (*p* < 0.05).

**Table 7:** Effects of stevioside supplementation on intestinal mucosal oxidative status of LPS-challenged broilers.

	Treatments ^2^					*p* value		
Items ^1^	CON	STE	LPS	LPS + STE	SEM	LPS	STE	Interaction
Jejunum								
MDA (nmol/mg protein)	1.91	1.60	2.41	2.13	0.14	0.001	0.043	0.909
T-AOC (mM/mg protein)	56.53 ^a^	55.83 ^a,b^	45.96 ^b^	56.32 ^a^	2.61	0.243	0.021	0.012
SOD (U/mg protein)	35.54	35.03	27.72	36.74	2.10	0.219	0.092	0.062
CAT (U/mg protein)	3.45	4.37	2.49	3.27	0.39	0.016	0.043	0.856
GSH-Px (U/mg protein)	12.39	19.22	6.53	12.97	1.25	<0.001	<0.001	0.891
Ileum								
MDA (nmol/mg protein)	1.71 ^b^	1.67 ^b^	2.67 ^a^	1.90 ^b^	0.14	0.001	0.019	0.033
T-AOC (mM/mg protein)	46.62	44.46	33.99	40.54	2.33	0.002	0.365	0.088
SOD (U/mg protein)	22.02 ^a^	21.92 ^a^	16.91 ^b^	23.39 ^a^	1.15	0.136	0.014	0.011
CAT (U/mg protein)	3.24	3.13	2.61	2.65	0.15	0.002	0.824	0.630
GSH-Px (U/mg protein)	10.88	12.99	6.18	7.18	1.14	<0.001	0.261	0.685

^1^ MDA, malondialdehyde; T-AOC, total antioxidant capacity; SOD, superoxide dismutase; CAT, catalase; GSH-Px, glutathione peroxidase; ^2^ CON, non-challenged broilers fed a basal diet; STE, non-challenged broilers fed a basal diet supplemented with 250 mg/kg stevioside; LPS, LPS-challenged broilers fed a basal diet; LPS+STE, LPS-challenged broilers fed a basal diet supplemented with 250 mg/kg stevioside; SEM, standard error of mean; ^a,b^ Means (*n* = 6) with different letters within a row are significantly different (*p* < 0.05).

**Table 8:** Effects of stevioside supplementation on antioxidant gene expression in the intestinal mucosae of LPS-challenged broilers.

	Treatments ^2^					*p* value		
Items ^1^	CON	STE	LPS	LPS + STE	SEM	LPS	STE	Interaction
Jejunum								
*SOD1*	1.00	1.19	0.76	0.98	0.06	0.001	0.002	0.739
*SOD2*	1.00 ^a^	0.97 ^a^	0.71 ^b^	0.95 ^a^	0.05	0.019	0.029	0.008
*CAT*	1.00	1.10	0.60	0.88	0.07	<0.001	0.012	0.211
*GPX1*	1.00	1.38	0.61	1.05	0.13	0.020	0.009	0.795
*Nrf2*	1.00 ^a^	0.99 ^a^	0.61 ^b^	0.95 ^a^	0.06	0.008	0.030	0.028
*HO-1*	1.00	1.12	0.50	0.71	0.07	<0.001	0.052	0.594
Ileum								
*SOD1*	1.00	1.17	0.72	1.01	0.07	0.005	0.004	0.436
*SOD2*	1.00	1.05	0.98	1.12	0.06	0.690	0.107	0.427
*CAT*	1.00	1.36	0.59	0.95	0.09	<0.001	<0.001	0.968
*GPX1*	1.00	1.34	0.98	1.39	0.19	0.969	0.086	0.877
*Nrf2*	1.00	1.34	0.94	1.30	0.15	0.745	0.040	0.946
*HO-1*	1.00	1.34	0.45	0.88	0.08	<0.001	<0.001	0.593

^1^*SOD1*, superoxide dismutase-1; *SOD2*, superoxide dismutase-2; *CAT*, catalase; *GPX1*, glutathione peroxidase 1; *Nrf2*, nuclear factor-erythroid 2-related factor 2; *HO-1*, heme oxygenase-1; ^2^ CON, non-challenged broilers fed a basal diet; STE, non-challenged broilers fed a basal diet supplemented with 250 mg/kg stevioside; LPS, LPS-challenged broilers fed a basal diet; LPS + STE, LPS-challenged broilers fed a basal diet supplemented with 250 mg/kg stevioside; SEM, standard error of mean; ^a,b^ Means (*n* = 6) with different letters within a row are significantly different (*p* < 0.05).
